# Barriers and facilitators to using a web-based tool for diagnosis and monitoring of patients with depression: a qualitative study among Danish general practitioners

**DOI:** 10.1186/s12913-018-3309-1

**Published:** 2018-06-27

**Authors:** Mette Daugbjerg Krog, Marie Germund Nielsen, Jette Videbæk Le, Flemming Bro, Kaj Sparle Christensen, Anna Mygind

**Affiliations:** 1 Research Unit for General Practice & Section for General Medical Practice, Department of Public Health, Aarhus University, Bartholins Allé 2, 8000 Aarhus C, Denmark; 2 Research Unit for General Practice, University of Southern Denmark, J. B. Winsløws Vej 9, 5000 Odense C, Denmark

**Keywords:** Telemedicine, eHealth, General practice, Primary health care, Depression, Major depression inventory, Psychometrics, Qualitative, Denmark, Determinants of behaviour

## Abstract

**Background:**

Depression constitutes a significant part of the global burden of diseases. General practice plays a central role in diagnosing and monitoring depression. A telemedicine solution comprising a web-based psychometric tool may reduce number of visits to general practice and increase patient empowerment. However, the current use of telemedicine solutions in the field of general practice is limited. This study aims to explore barriers and facilitators to using a web-based version of the Major Depression Inventory (eMDI) for psychometric testing of potentially depressive patients in general practice.

**Methods:**

Semi-structured individual interviews were conducted with nine general practitioners (GPs) from eight general practices in the Central Denmark Region. All interviewees had previous experience in using the eMDI in general practice. Determinants for using the eMDI were identified in relation to the GPs’ capability, opportunity and motivation to change clinical behaviour (the COM-B system).

**Results:**

Our results indicate that the main barriers for using the eMDI are related to limitations in the GPs’ opportunity in regards to having the time it takes to introduce change. Further, the use of the eMDI seems to be hampered by the time-consuming login process. Facilitating factors included behavioural aspects of capability, opportunity and motivation. The implementation of the eMDI was facilitated by the interviewees’ previous familiarity with the paper-based version of the tool. Continued use of the eMDI was facilitated by a time-saving documentation process and motivational factors associated with clinical core values. These factors included perceptions of improved consultation quality and services for patients, improved possibilities for GPs to prioritise their patients and improved possibilities for disease monitoring. Furthermore, the flexible nature of the eMDI allowed the GPs to use the paper-based MDI for patients whom the eMDI was not considered appropriate.

**Conclusions:**

Implementation of a telemedicine intervention in general practice can be facilitated by resemblance between the intervention and already existing tools as well as the perception among GPs that the intervention is time-saving and improves quality of care for the patients.

## Background

The global burden of mental diseases is increasing. Depression is currently the most common mental disorder, affecting approximately 300 million people worldwide according to the World Health Organization [1]. General practice plays a central role in the diagnosis and monitoring of depression [2, 3], and many general practitioners (GPs) use psychometric tools for assessment.

A range of rating scales and psychometric tools are used by GPs worldwide for diagnosis and monitoring of depression [4]. The most frequently used tool in Denmark is the Major Depression Inventory (MDI), and Danish GPs are remunerated for undertaking psychometric testing [2]. The MDI is a self-report questionnaire in which the patient scores the frequency of his or her symptoms during the last 2 weeks [4]. It explores the ICD-10 criteria for depression in a questionnaire form. On the basis of the patient’s individual symptom score, the GP calculates an overall MDI summary score, which serves as a measure of depression severity (mild, moderate or severe) and for monitoring changes over time.

Telemedicine has been introduced as a new tool to meet the current challenges of the increasing prevalence of depression and chronic diseases [5] and has become a political priority worldwide [6]. Telemedicine is a concept around which many different definitions prevail. As a result, the WHO has adopted the following broad definition of telemedicine, which we subscribe to in our study:

*“The delivery of health care services, where distance is a critical factor, by all health care professionals using information and communication technologies for the exchange of valid information for diagnosis, treatment and prevention of disease and injuries, research and evaluation, and for the continuing education of health care providers, all in the interests of advancing the health of individuals and their communities”* [6].

The implementation of telemedicine on a wider scale has been suggested as a way to reduce the number of healthcare visits and provide empowerment of patients. Despite great expectations, clear evidence of economic benefits of telemedicine is lacking, e.g. due to limited implementation of large-scale projects [6–8].

To increase the use of a specific telemedicine tool (WebPatient) in Danish general practice, the Danish Ministry of Health launched a new initiative in 2015 aiming to implement WebPatient in Danish general practice over a three-year period [9]. In this system, the GP can request different web-based tests including the eMDI. The requested test is sent to the patient’s mailbox through an electronic link with a following electronic instruction on how to fill it in. The overall score is then automatically returned to the GP’s electronic patient record. The content in the paper-based MDI and the eMDI is identical, and the GP can freely choose between the two.

When the eMDI was launched, it was expected to replace the paper-based MDI and save visits to the GP [10]. The eMDI has, however, so far not been implemented to the extent that was originally expected, and the reasons behind have not been systematically explored. This is the case for many new telemedicine initiatives [6].

### Theoretical frame

The Behaviour Change Wheel offers a comprehensive structured framework for guiding the design and evaluation of interventions and is based on the COM-B model [11]. COM stands for the three behavioural factors Capability, Opportunity and Motivation, whereas B is short for Behaviour. Capability is understood as skills, strength, stamina (both physical and mental) and knowledge. Opportunity involves factors outside the individual (time, organizational structures, legislation, etc.). Motivation can be reflective (goals, values and conscious decision-making) or automatic (emotional response and habitual processes) [11]. The COM-B analysis aims to explore how a specific behaviour is influenced by the COM-B factors, thereby revealing how behaviour is promoted or impeded by the individual’s capabilities, opportunities and motivation.

### Aim of the study

This study aims to explore facilitators and barriers to using the eMDI in psychometric testing of patients with symptoms of depression in Danish general practice by gaining insight into the behaviour of GPs who use the tool and factors affecting their behaviour in terms of capability, opportunity and motivation.

## Methods

### Design

We conducted a qualitative study by performing semi-structured individual interviews with GPs to explore their views on and experiences with the eMDI.

### Recruitment

To include GPs with sufficient experience of using the eMDI, we adopted a purposeful sampling strategy [12] applying the inclusion criterion that interviewees must be current users of the eMDI. We made use of information about their current use of the eMDI from MedCom (a non-profit organization, which monitors the use of eMDIs by each Danish general practice in WebPatient) to ensure that only GPs from practices with significant usage were invited. We invited 35 GPs from these MedCom lists. Additionally, we invited 15GPs from development practices (i.e. practices participating in small-scale pilot-testing of quality development projects before large-scale implementation) in the Central Denmark Region [13].

During February and March 2017, a total of nine interviewees were recruited (six recruited from MedCom lists and three from development practices) (Table 1). Six interviewees were male, and three were female. The age of the interviewees ranged from 44 to 67 years. Eight interviewees worked in partnership practices, and one worked in a solo practice.

**Table 1:** Overview of interviewees

Interviewee	Age (years)	Working experience in general practice (years)
I1	50–59	> 25
I2	60–69	> 30
I3	40–49	> 5
I4	40–49	> 10
I5	40–49	< 5
I6	50–59	> 15
I7	50–59	> 10
I8	50–59	> 10
I9	60–69	> 15

### Data collection

Interviews were conducted in March and April 2017 in a setting chosen by the interviewees. Seven interviews took place in the interviewee’s own clinic, and two interviewees preferred a telephone interview.

The semi-structured interview guide included questions about the interviewees’ specific behaviour, such as how, when and for whom they use the web-based eMDI. Questions were related to aspects of capability, opportunity and motivation as well as the interviewees’ general experiences of problems with and benefits of using the eMDI in their daily work (Table 2). The interview guide was tested by performing pilot interviews with two GPs who were experienced users of the eMDI but were not enrolled in the study.

**Table 2:** Overview of interview guide

Behavioural factor	Theme	Sub-theme	Example of probing question
Capability	Psychological capability	Technical skills in relation to the eMDI	*Did you feel confident in using the electronic form when using it for the first time? Was it difficult? Easy? Do you feel more confident when using it now?*
Opportunity	Social opportunity	Organisational setting	*Can you describe the process of implementing the eMDI in your practice? How did you get started as a practice?*
	Physical opportunity	Aspects of time and resources in the clinic	*How would you describe the settings and opportunities for embedding the eMDI here in your practice? Which challenges have you experienced? Which do you still experience?*
Motivation	Reflective motivation	Feelings of autonomy and competence in using the eMDI	*When you first started using the eMDI, did you feel that it was imposed on you? Or did you start using it out of your own interest? Did you feel confident about using the eMDI?*
		Values	*Which pros and cons of the eMDI do you see as compared to the paper-based version?* *Which considerations do you take into account when choosing between the two?* *Has the eMDI in any way influenced the relationship with your patient?*
		Attitude towards telemedicine	*How do you feel about the increasing use of telemedicine in general practice?*
		Prioritising tool	*For whom and when do you typically use the eMDI? And the paper-based MDI?*
	Automatic motivation	Habits and routinisation	*How often do you use the eMDI instead of the paper-based MDI? What could make you choose the eMDI even more frequently?*

All interviews were recorded and transcribed verbatim, and additional field notes were written down immediately after each interview. As a means to secure validity of the interviews, refine the interviewguide and to increase information power [14], MDK and AM continuously discussed transcripts during data collection. Sampling ceased when new barriers and facilitators stopped emerging and we found to have enough information to adequately answer the research question. The interviews lasted 20–60 min with an average length of 45 min.

### Analytical procedure

Following the hermeneutic approach [15], the analysis switched between looking at the full interview as a single meaningful unit and at smaller units of meaning within the text. First, the transcripts were read thoroughly by MDK to get an overall impression of the material. Meaningful units were identified and coded thematically in accordance to the components in the COM-B model. This initial thematic coding was conducted by MDK and AM. The coding process was discussed in the research group consisting of general practitioners (FB, KSC), researchers with experience in the field of telemedicine (MDK, KSC, MGN), qualitative methods (MDK, FB, AM, JVL) and application of the COM-B model for research purposes (FB, JVL, AM). This iterative process led to agreement on the most important sub-themes within the components of the COM-B model. These sub-themes were further classified as either barriers or facilitators for the use of the eMDI.

Throughout the analysis, the researchers’ pre-understandings were challenged by switching between open and focused coding of data. Thus, the three behavioural components capability, opportunity and motivation formed the basis of the focused coding process, while sub-themes emerged more freely through open coding. Data were analysed using NVivo 11 [16].

## Results

The interviewees have different experiences with the use of the eMDI in daily clinical practice. Variations include the extent to which the eMDI has become part of their day-to-day routine and how, when and for whom the eMDI is used. Table 3 shows an overview of the identified facilitators and barriers for the use of the eMDI, as they emerged from the analysis. The most dominant themes, emerging in all or most interviews, are also presented.

**Table 3:** Facilitators and barriers emerging from the analysis

Facilitators	
*COM-B component*	*Sub-theme*
Capability	Familiarity with the paper-based MDI^a^
Opportunity	Easy documentation process^a^
Motivation	Not fewer, but better consultations^a^
Motivation	Improved services for patients
Motivation	Improved prioritisation between patients
Motivation	Improved patient monitoring^a^
Motivation	Flexible use for selected patients
Barriers	
*COM-B component*	*Sub-theme*
Opportunity	Resource demanding introduction of change^a^
Opportunity	Time-consuming login to the system

^a^Themes emerging in most or all interviews

The barriers and perceived facilitators related to the use of the eMDI will be described in the following with reference to the three behavioural components of the COM-B model: *capability*, *opportunity* and *motivation*. It will be emphasized whether and how elements belonging to the three components occur as barriers or facilitators in reaching the target behaviour, understood as use of the eMDI. When relevant, a distinction will be made between behaviour in relation to diagnostics and monitoring.

### Capability

#### Familiarity with the paper-based MDI

Many of the interviewees mention similarity between the traditional paper-based MDI and the eMDI as a facilitator in the implementation of the eMDI. A frequent user of the eMDI explains:

*“If you had to start from scratch on both the electronic part and the form itself, it would have been more difficult. But as it’s a natural development, I think it was quite (…), I don’t think it was difficult.”* (I1).

Another interviewee agrees and hypothesises that inexperience with using the MDI might hamper the routine use of the eMDI:

*“Then it’s like: ‘Am I doing it right? Do I interpret it in the right way?’. And then you might end up being critical about it. And in my opinion that’s wrong. Because it’s about something else, it could be that you are insecure in another way.”* (I8).

She elaborates on how her previous experience with the MDI has made the transition from the paper version to the electronic version easier. Previous experience with the MDI thus appears to serve as a facilitator in the process of embedding the eMDI into the GPs’ daily routines.

### Opportunity

#### Easy documentation process

All interviewees perceived the eMDI as a timesaver compared to the paper version since the latter requires printing, scanning and entering the results from the forms into the laboratory system of the clinic. Thus, they explain how the introduction of the eMDI has made the process of documenting the patients’ scores easier. For many of the interviewees, this was a significant facilitator in their use of the eMDI.

#### Resource demanding introduction of change

Even though the eMDI *gives* the interviewees more time for other tasks, it also *takes* time to change one’s routines. The interviewee who uses the eMDI the least expresses this duality:

*“If you want change to happen sooner and more [efficiently], then you have to hire twice as many GPs so we won’t be as busy as we are now… because from when we walk into our clinic in the morning and until we leave again, we usually work like little beasts. And then it’s just, then you draw on your routines. So it takes time to create change.”* (I4).

The extra effort that it takes to change routines acts as a significant barrier in the introduction of change. As a reflection of this, the interviewees have embedded the use of the eMDI into their daily routines at various degrees. One interviewee who rarely uses the paper form anymore says the following about choosing the eMDI:

*“It has become an ingrained part of my routines […] So it’s a natural part of my routines now. So from being something I remembered to use a lot of the time in the beginning, it is now entirely what I use.”* (I7).

This quote describes what happens when behaviour changes from being something you make yourself do into something you just do.

#### Time-consuming login to the system

All interviewees agree that the eMDI is easy to use, and they experience no challenges in meeting the technical capabilities required, which is partly due to their familiarity with using web-based laboratory tests as an integrated part of their daily work. Despite this familiarity, some of the interviewees experience a barrier in the time-consuming process of logging on to the patient’s account during the consultation. The login process requires the patient to enter his or her social security number, a password and then a specific numbercode combination. One interviewee explains why he does not use the eMDI for the patients he assesses to be unable to fill it in at home:

*“It would require the patient to log on to the system with NemID [Danish online login solution] during the consultation. Then half of the consultation would be spent, and I don’t have time for that. So I will probably use the other solution [the paper-based MDI].”* (I5).

Thus, aspects revolving around time and efficiency mostly appear to promote the GPs’ use of the eMDI. Though, for some interviewees, these aspects have worked as an unfeasible barrier because of lack of time to change their routines and experiences with the eMDI as being too time-consuming when filled in during the consultation.

### Motivation

#### Not fewer, but better consultations

All interviewees use the eMDI as a supplement to the face-to-face encounter with the patient since dialogue is essential when evaluating the mental condition of the patient. Thus, every completed form, both the paper-based and the web-based version, is followed by a consultation. Only in a few cases (if the patient is in recovery or has a low MDI score), the interviewees replace the face-to-face consultation with an e-mail or a telephone consultation.

Furthermore, the personal contact and dialogue between GP and patient is an essential value for the interviewees. One interviewee, who is generally worried about the increasing number of forms and figures in general practice, sees greater advantages in the eMDI compared to the paper-based MDI because the eMDI releases time in the consultation. When one of the interviewees is asked if the number of visits by patients with depression has changed since he started using WebPatient, he reflects:

*“When it comes to people with depression, the number [of visits] is the same, but one could imagine that it would be possible to save some visits when it comes to controls of blood pressure. But not with depression.”* (I5).

The time released by the eMDI for dialogue in the consultation is welcomed by the interviewees. It is seen as improved quality of the consultations and thereby improved patient treatment, and this is mentioned as one of the greatest facilitators in the acceptance of the tool. At the same time, the interviewees express that the eMDI has this advantage only if followed by a face-to-face consultation. Thus, the eMDI is seen as a supplement to (rather than a replacement of) an actual consultation. For several of the interviewees, it is also common practice to fill in the first form (paper- or web-based version) together with the patient during the consultation. They explain that, by providing an oral instruction, they attempt to improve the quality of the test result since misunderstandings on the patients’ part can then be minimised.

#### Improved services for patients

Some interviewees report that the use of the eMDI may provide more well-being for the patients because they can fill it in at home at their own pace without having to take the trip down to the clinic every time. The eMDI is thus regarded as a more accessible solution for some, e.g. for patients with a full-time job and a low MDI score; the following consultation can then be delivered as an e-mail consultation or by telephone.

#### Improved prioritisation between patients

With an increasing demand from people seeking help for mental issues, the eMDI is, by some interviewees, welcomed as a tool for prioritising the GP’s time. This advantage is described by one of the interviewees:

*“If you can sense that they [the patients] are not suicidal, and you’re thinking ‘this can be put off for a couple of days’, and you don’t have a spare moment in your schedule here and now, then I often say to them ‘now you will get this form emailed to you, which I want you to fill in’. If I then receive a form the same night and it looks just terrible, then I can get hold of the person urgently and not postpone it, right.”* (I9).

A few interviewees, who see a lot of patients with depressive symptoms in their practice, mention that they have great use of the eMDI, not just for prioritisation purposes but also because it enables them to give an immediate response without seeing the patient on the same day. This makes the patient feel taken care of, and the eMDI has increased the accessability of the services provided by the GPs. Accordingly, the GPs with large flows of patients with depression mention prioritisation as a facilitating factor in their day-to-day use of the eMDI.

#### Improved patient monitoring

One interviewee, who mainly uses the eMDI for monitoring purposes explains that using the eMDI, has improved his monitoring competencies:

*“Our monitoring of these patients [with depression] gets better because we are capable of following their development over time, which we have neglected before (…). We are [now] better at meeting the demands set up for us as GPs”.* (I5).

Overall, the interviewees perceive the improved possibilities for monitoring as the greatest advantage of the eMDI, and this has contributed significantly to their choice of initiating the use of the tool. Correspondingly, several interviewees state to have used the tool for monitoring purposes rather than for diagnostic purposes. One interviewee also mentions how the availability of the eMDI has broadened her general use of the MDI. While her use of the paper-based test was limited to diagnostics before the introduction of the eMDI, she now also includes monitoring of those of her patients who are asked to complete the eMDI.

#### Improved use of consultation time

Some interviewees mention that the eMDI offers better preparation before a consultation. They use the data provided by the eMDI as a starting point for the dialogue with the patient since they have already seen the results of the test before the consultation begins. In contrast, some interviewees explain how, when using the paper-based MDI, they use part of the consulation time for creating an overview of the test results. Improved competencies in the shape of improved preparation and enhanced monitoring are mentioned as facilitating factors for both the initiation and the continued use of the eMDI.

#### Flexible use for selected patients

All interviewees find that the eMDI is not appropriate for all patients. The possibility for the interviewees to still use the paper form for certain patients is perceived as a facilitator for implementing the eMDI. The elderly patients are deselected because of their perceived inexperience with IT and electronics. Also, some interviewees point out that patients with language and reading difficulties need help from their GP to fill in the form. In these cases, the interviewees usually choose the paper form since it does not require login to the patient’s system account. Another subgroup of patients for whom the paper version is sometimes considered favourable is the severely ill patients who suffer from suicidal thoughts and lack of the energy to cope with even simple daily tasks. For these patients, the GPs find that it can be an inconceivable task for the patients to fill in a web-based form. Therefore, they prefer to use the paper form or to enter the patient’s answers in the eMDI for him or her. One interviewee summarises why the eMDI is not appropriate for all patients:

*“It requires a patient who has some competencies, who feels like trying it and who isn’t afraid of trying it out because we don’t have the time to take them by the hand and show them step by step how to do it. I understand that there is an online explanation of the form, but this also requires that the patient is capable of reading it. So it takes a resourceful patient. And then it’s always, when we get to the psychometric tests, then it’s always a consideration (…) are people too ill to manage to get it done and is it necessary to have them here in the clinic instead”* (I3).

The interviewees generally imagine that they will predominantly use the eMDI in the future, but they also find that there will always be a few patients in their clinic for whom the paper form will be more appropriate.

## Discussion

### Main findings

Our results show that the barriers for using the eMDI mainly relate to the behavioural aspects of opportunity, whereas facilitating factors mainly include aspects of capability and opportunity, and to a lesser degree motivational factors. Thus, the introduction of change takes time and the routine use of the eMDI can be hampered by the time-consuming login process. The initial use of the eMDI was facilitated by the fact that the interviewees were already familiar with the paper-based MDI. Continued use of the eMDI was facilitated by a time-saving documentation process and motivational factors associated with clinical core values such asimproved consultation quality and services for patients and improveddisease monitoring.

### Limitations of the study

The practices involved in this study were chosen among practices where the eMDI was relatively well implemented and where GPs thus took a relatively positive attitude towards the tool. Therefore, the barriers related to initiation of eMDI use are not fully explored. Other barriers could be general reluctance towards rating scales or IT, e.g. negative attitudes to electronic solutions and concerns about privacy, as found in other studies of implementation of telemedicine in primary care [17–19]. However, this limitation was chosen in order to explore the GPs’ lived experiences with initiation of use and routine use of the eMDI.

When judging the appropriateness of a sample size, the risk of undermining the credibility of the findings by a small sample size must be weighed against the risk of having too much data to complete a detailed analysis [20, 21]. Despite the relatively small number of GPs, we believe that our data material covers a range of important barriers and facilitators towards eMDI although we cannot claim to have covered all aspects of potential barriers and facilitators. Inclusion of a larger number of GPs and GPs with lower eMDI implementation rates might also have elicited potential differences across other factors, e.g. explored the influence of GPs’ IT skills on eMDI use. Such trends in eMDI implementation according to the GPs’ age, gender, working experience and IT familiarity cannot be explored in the current study.

The use of the semi-structured interview has some limitations. While dialogue is an essential element of this methodology, the interaction taking place between interviewer and interviewee can affect the interviewee’s answers [22]. Thus, difficulties with securing reliability is an inherent issue in qualitative research. Also, when following the hermeneutic approach used in this study, the results will inevitably be influenced by the researchers’ pre-understandings of the explored subject [15]. Pre-understandings and findings were, therefore, continuously reflected upon and discussed within the research team.

### Implications of findings in context of existing research

We found that the use of the eMDI was facilitated by the existing familiarity with the paper-based form already well known and implemented in Danish general practice. This is in line with the findings of a review on the implementation of eHealth, which underlines the importance of an outer context, e.g. in terms of recognised standards [19]. This familiarity with MDI can also be understood as a way of improving the socio-technical ‘fit’ (i.e. how well the electronic solution fits with the social features of general practice), which has previously been found to influence the implementation of telemedicine in general practice [17].

In our study, we also found that a time-consuming login process was a barrier in some consultations, specifically because consultations are usually limited to 10–15 min. The time that it takes to introduce change in the practice and change your own habits was also mentioned as a significant barrier. The importance of time factors is supported by other studies [17–19].Thus, barriers towards utilisation revolved more around the physical opportunity related to the available time and resources than around the physicians’ capabilities since the simplicity of the eMDI made no significant demands on the GPs technical capabilities.

However, the time and resources released by using the eMDI was also identified as one of the prominent facilitators that could influence the use of it. This duality with regard to time has also been mentioned in another study in the general practice setting, where time and resources are under pressure [23]. It is therefore important, when designing interventions to support implementation of new activities such as the eMDI, to acknowledge that time spent to introduce it is a barrier, while time saved once it is routinized is a facilitator. Our study reports a new finding; the benefit of the web-based solution for prioritisation of patients. This derivative effect seems to be especially welcomed by those interviewees who see many patients with depression because the web-based solution enables them to provide immediate care for the less acute patients without seeing them on the same day.

The eMDI is perceived to enhance the quality of consultations and thereby, assumably, improve patient outcomes, most importantly by providing more time for targeted dialogue and therapy during the consultation. The perception that improved quality of care and clinical relevance of content (e.g. activation of patients and better clinical assesments made by GPs) are facilitating components in the implementation of telemedicine in primary care is supported by a range of other studies [17, 18, 24, 25].

Some studies have found that telemedicine may alter the traditional doctor-patient relation because part of the human contact is replaced by electronic contact [26]. The GPs’ concerns about this has also been found to hamper implementation of telemedicine [17, 19]. In our study, we found that the implementation of the eMDI was facilitated by the flexible nature of the instrument, which allowed the GPs to adapt their use to the individual patients. This flexibility, where GPs can choose not to use the electronic solution for patients who are vulnerable or who have limited language or IT skills, may help overcome barriers concerning less doctor-patient interaction. Furthermore, it is wellknown that e-health solutions are not applicable to all patients [27]. Generally, the interviewees found the web-based solution suitable for the young and resourceful patients with adequate IT skills and reading abilities. A few other studies found similar results in regard to age and IT skills [23, 28]. At the same time, studies in the field of telemedicine generally highlight the benefit of increased accessability of health services for marginalised groups, such as immigrants and low-income groups [24, 29]. This is in contrast with the findings in this study as the interviewees usually chose the traditional paper-based solution for these patients since they often have less resources and lower reading abilities. In our study, the ones who benefit from the increased accessability are the patients who have difficulties with getting time off work to go to the clinic.

### Recommendations for future research

To obtain more knowledge about the facilitators and barriers of eMDI implementation in general practice, further studies should explore the experiences among non-users, e.g. by interviewing GPs who have not to implemented the eMDI at all or only to a low degree. Additionally, ongoing monitoring and evaluation of the eMDI should be conducted in order to ensure further uptake, including evaluation of the login process. To ensure full insight into the facilitators and barriers concerning the use of the eMDI and to make adequate recommendations, the perspectives of the patients should also be visited in further studies.

## Conclusions

Our study suggests that uptake and continuos use of electronic tools in general practice can be improved through 1) incorporation of well-known features of other tools to reduce unfamiliarity in both content and packaging, 2) emphasis on positive effects of the tool related to core GP values (e.g. enhanced quality of care and accommodation of patient needs), and 3) allocation of adequate time and resources to GPs to allow them to adapt the new tool and change their routines. Implementation of telemedicine interventions in general practice could be supported by taking these aspects into account.

## Abbreviations

eMDI: Electronic version of the MDI
GP: General practitioner
I: Interviewee
MDI: Major Depression Inventory
